# Wheat, Rye, and Barley Genomes Can Associate during Meiosis in Newly Synthesized Trigeneric Hybrids

**DOI:** 10.3390/plants10010113

**Published:** 2021-01-07

**Authors:** María-Dolores Rey, Carmen Ramírez, Azahara C. Martín

**Affiliations:** 1 Agroforestry and Plant Biochemistry, Proteomics and Systems Biology, Department of Biochemistry and Molecular Biology, University of Córdoba, 14014 Córdoba, Spain; b52resam@uco.es; 2 Institute for Sustainable Agriculture (IAS-CSIC), Department of Plant Genetic Improvement, 14004 Córdoba, Spain; carmenramirez@ias.csic.es; 3 Crop Genetics Department, John Innes Centre, Norwich NR4 7UH, UK

**Keywords:** wheat, *Hordeum chilense*, *Triticeae*, WGD (whole genome duplication), hybridization, meiosis, recombination, synapsis, GISH

## Abstract

Polyploidization, or whole genome duplication (WGD), has an important role in evolution and speciation. One of the biggest challenges faced by a new polyploid is meiosis, in particular, discriminating between multiple related chromosomes so that only homologs recombine to ensure regular chromosome segregation and fertility. Here, we report the production of two new hybrids formed by the genomes of species from three different genera: a hybrid between *Aegilops tauschii* (DD), *Hordeum chilense* (H^ch^H^ch^), and *Secale cereale* (RR) with the haploid genomic constitution H^ch^DR (n = 7× = 21); and a hybrid between *Triticum turgidum spp. durum* (AABB), *H. chilense,* and *S. cereale* with the constitution ABH^ch^R (n = 7× = 28). We used genomic *in situ* hybridization and immunolocalization of key meiotic proteins to establish the chromosome composition of the new hybrids and to study their meiotic behavior. Interestingly, there were multiple chromosome associations at metaphase I in both hybrids. A high level of crossover (CO) formation was observed in H^ch^DR, which shows the possibility of meiotic recombination between the different genomes. We succeeded in the duplication of the ABH^ch^R genome, and several amphiploids, AABBH^ch^H^ch^RR, were obtained and characterized. These results indicate that recombination between the genera of three economically important crops is possible.

## 1. Introduction

Polyploidization or whole genome duplication (WGD) has an important role in evolution and speciation, and it is now accepted that all seed plants and angiosperms have experienced multiple rounds of WGD during their evolutionary history [1]. Polyploids, species with more than a diploid complement of chromosomes, are grouped in two main categories: autopolyploids, which arise from intraspecific genome duplication, and allopolyploids, in which WGD is coupled with interspecific hybridization. Interestingly, many of the world’s most important crops, including wheat, rapeseed, sugarcane, and cotton, are relatively recent allopolyploids. This is not a coincidence; allopolyploids often show higher adaptability (they clearly show better tolerance to abiotic stresses), can grow over larger geographical areas, and show better adaptation to the local environment than their diploid progenitors [2]. The reasons for this particular success are not clear, but they are probably the result of multiple evolutionary processes such as rapid genome organisation, fractionation, gene conversion, transgressive gene expression alteration, and sub- and/or neofunctionalization of duplicate genes [1]. However, despite their obvious advantages in adaptation, newly formed allopolyploids face the challenge of organizing two or more genomes (subgenomes) that have evolved independently, within a single nucleus. Many will be the challenges, but probably, the biggest of all will be meiosis. Meiosis is the specialized cell division that generates haploid gametes for sexual reproduction. During meiosis, homologous (identical) chromosomes synapse along their length and recombine, leading to novel combinations of parental alleles. At least one crossover (CO) needs to be formed between every pair of homologs to ensure accurate chromosome segregation and balanced gametes. In newly formed allopolyploids, apart from the two identical homologs present in diploid species, there are also very similar chromosomes (homeologs), which will complicate the process of recognition and synapsis between homologs. As a consequence, allopolyploidization is frequently accompanied by irregular meiosis, unbalanced gametes, and sterility. Only the few allopolyploids that manage to overcome this initial bottleneck of instability, will be able to be established and join the evolutionary fight as efficient competitors of their diploid relative [2].

*Triticeae*, an economically important tribe which includes major crop genera such as wheat, barley, and rye, has been extremely successful in taking advantage of this speciation mechanism. The *Triticeae* tribe within the grass family *Poaceae* (*Gramineae*) includes about 360 species, from which only 80 species are diploid, being the majority allopolyploids derived from interspecific and intergeneric hybrids [3]. Moreover, two synthetic allopolyploids have been obtained by intergeneric hybridization, followed by chromosome doubling of the hybrids, which showed enough fertility to be considered as crops: *×Triticosecale*, an amphiploid between wheat and rye [4]; and *×Tritordeum*, an amphiploid between the wild barley *Hordeum chilense* Roem. et Schult. and wheat [5]. This huge capacity for interspecific hybridization among the *Triticeae* has been used by breeders for a long time to transfer interesting traits from related or wild species into crops, particularly into modern wheat [6,7,8,9,10,11,12,13,14,15,16]. Because of their agricultural importance, the *Triticeae* species have been bred intensively for the past hundred years, resulting in massive improvements in yield and quality. However, this success has been associated with a narrowing of the available genetic diversity within elite germplasm, and there is concern that the prospects for continued genetic gain are becoming increasingly limited [17]. Thus, both to better understand polyploidization and domestication and to facilitate the exchange of genetic material among species, it is very important to study the genomic relationship among these species and analyze the meiotic behavior of their chromosomes when they are put together within the same nucleus.

In this study, we report the production of two new intergeneric hybrids formed by the genomes of species from three different genera inside the *Triticeae* tribe: a trihybrid between *Aegilops tauschii* (2n = 2× = 14, DD), *H. chilense* (2n = 2× = 14, H^ch^H^ch^), and *S. cereale* (2n = 2× = 14, RR) with the haploid genomic constitution H^ch^DR (n = 3× = 21 chromosomes); and a trihybrid between *T. durum* (2n = 4× = 28, AABB), *H. chilense,* and *S. cereale* with the haploid genomic constitution ABH^ch^R (n = 4× = 28 chromosomes). *Ae. tauschii*, the diploid progenitor of the D genome of hexaploid wheat (*T. aestivum*, genomes AABBDD) [18], is an important source of genetic variability for bread wheat. The hybridization between tetraploid wheat and *Ae. tauschii* occurred only ~8000 years ago, and just a few accessions might have been involved in this event. Thus, we find a much greater genetic diversity among wild wheat progenitors and closely related cereal species than within the cultivated wheat gene pool. *H. chilense* Roem. et Schultz. is a diploid wild barley native to Chile and Argentina [19], which possesses some useful traits for wheat breeding such as drought and salt tolerance [7,20], resistance to pests and diseases [21], and a high seed carotenoid content [22]. In this work, we use genomic *in situ* hybridization (GISH) to establish the chromosome composition of the new hybrids and to study their meiotic behavior at metaphase I. Multiple chromosome associations at metaphase I, indicating the possibility of recombination between the different genomes, were observed, particularly in the H^ch^DR hybrid. We also use immunolocalization of key meiotic proteins to establish the level of synapsis among the different genomes, and finally, we duplicate the ABH^ch^R hybrid to obtain the corresponding diploid amphiploid, which we further analyze.

## 2. Results

### 2.1. Production, Chromosome Constitution, and Morphology of the Trigeneric Hybrids and Amphiploids

#### 2.1.1. Hybrid H^ch^DR

**Figure 1:**
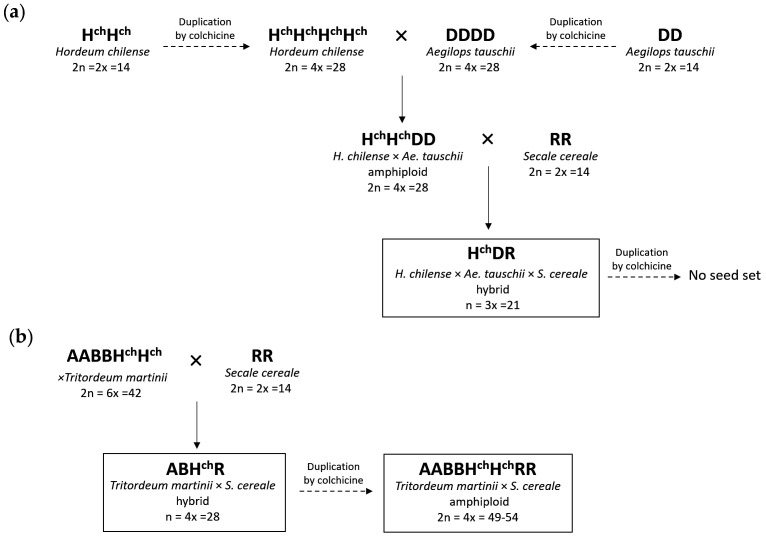
Breeding scheme followed to obtain the trigeneric hybrids and corresponding amphiploids used in this work. (**a**) Production of the hybrid H^ch^DR between *Hordeum chilense* (barley), *Aegilops tauschii,* and *Secale cereale* (rye). (**b**) Production of the hybrid ABH^ch^R between *Triticum turgidum* (durum wheat), *H. chilense,* and *S. cereale*, and the corresponding amphiploid.

Both *H. chilense* accession H7 (2×) and *Ae. tauschii* (2×) were easily duplicated after colchicine treatment. Seeds collected after colchicine treatment were germinated and the chromosome number counted until three tetraploids from each genotype were identified. Tetraploid *H. chilense* was pollinated with tetraploid *Ae. tauschii* (4×). From about 100 florets pollinated, six adult H^ch^H^ch^DD hybrid plants were established after embryo rescue. Finally, *H. chilense* × *Ae. tauschii* hybrids were pollinated with rye (*S. cereale*), and out of about 200 florets pollinated, two adult hybrids H^ch^DR were recovered by embryo rescue (Figure 1a).

**Figure 2:**
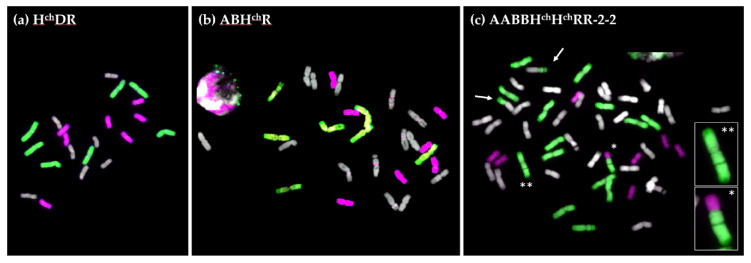
Root-tip metaphases of the trigeneric hybrids and amphiploid obtained in this work analyzed by GISH. (**a**) Hybrid H^ch^DR (n = 3× = 21) showing seven chromosomes of *Hordeum chilense* chromosomes (magenta), seven of rye (green), and seven of *Aegilops tauschii* (grey). (**b**) Hybrid ABH^ch^R (n = 4× = 28) using tritordeum HT474, with 14 chromosomes from durum wheat (grey), seven from *H. chilense* (magenta), and seven from rye (green). (**c**) Aneuploid amphiploid AABBH^ch^H^ch^RR-2-2 with 51 chromosomes. Two centromeric translocation between rye and one of the durum wheat chromosomes are indicated by an arrow. Another centromeric translocation between *H. chilense* and rye is marked with an asterisk and is enlarged. A reorganized rye chromosome with a NOR (nucleolar organizer region) in the long arm is indicated with two asterisks and is enlarged.

Root tip metaphase spreads were analyzed by multicolour GISH to verify the genome constitution of the trigeneric hybrid H^ch^DR (Figure 2a). Genomic DNA of *H. chilense* (H^ch^ genome) and rye (R genome) was used as probes, leaving *Ae. tauschii* (D genome) without labelling. The three recovered hybrids had 21 chromosomes, comprising seven chromosomes from *H. chilense* (in magenta), seven chromosomes from rye (in green), and seven chromosomes from *Ae. tauschii* (in grey) (Figure 2a), confirming that they were true trigeneric hybrids (n = 3× = 21).

**Figure 3:**
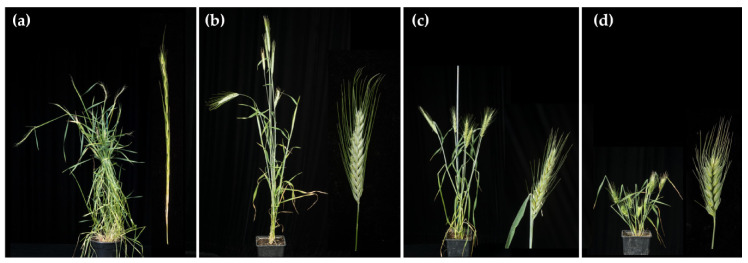
Adult plant and spike from (**a**) the H^ch^DR hybrid, (**b**) the ABH^ch^R hybrid, (**c**) the AABBH^ch^H^ch^RR-2-1 amphiploid, and (**d**) the AABBH^ch^H^ch^RR-1-1 amphiploid.

H^ch^DR hybrids showed vigorous vegetative growth and tillered profusely. They showed similar morphology to the female parent H^ch^H^ch^DD (*H. chilense* × *Ae. tauschii* hybrid) (Figure 3a), as already reported in another trigeneric hybrid where a different *H. chilense* accession was used [23]. The plants can grow vegetatively for years by cutting the developed spikes and allowing them to regrow new shoots, which is probably due to the *H. chilense* genome. The hybrid spike was thin and long with spikelets well separated along the rachis. All the florets were sterile.

#### 2.1.2. Hybrid ABH^ch^R

To produce the trigeneric hybrid between *T. turgidum*, *H. chilense,* and *S. cereale*, we used hexaploid *×Tritordeum martinii* lines HT377 (with the translocation T1RS·1BL) and HT474. Hexaploid tritordeum is the amphiploid between *H. chilense* (2n = 2× = 14; H^ch^H^ch^) and *T. turgidum* spp. *durum* (2n = 4× = 28; AABB); therefore, it is already a hybrid between barley and durum wheat. Tritordeum was crossed as the female parent with rye as the male parent. Around 100 florets were pollinated, of which five seeds were recovered, three using HT377 and two using HT474. Although the seeds were small and shriveled, they possessed enough endosperm to germinate and grow without the need of embryo rescue. These five seeds all developed well into adult ABH^ch^R hybrids (Figure 1b).

Root tip metaphase spreads were analyzed by multicolour GISH to verify the genome constitution of the trigeneric hybrids ABH^ch^R obtained (Figure 2b). Genomic DNA of *H. chilense* (H^ch^ genome) and rye (R genome) was used as probes, leaving *T. turgidum* (A and B genomes) without labelling. All recovered hybrids contained 28 chromosomes (Figure 2b), containing 14 chromosomes from the AB genome (Figure 2b in grey), seven from *H. chilense* (in magenta), and seven from rye (in green). This confirmed that they were true trigeneric hybrids (n = 4× = 28; ABH^ch^R).

The ABH^ch^R hybrids all showed similar morphology to the female parent (tritordeum AABBH^ch^H^ch^), with an erect vegetative development and a lower number of tillers than the H^ch^DR (Figure 3b). Spike morphology was also very similar to tritordeum. This hybrid displayed a morphology more characteristic of a crop and not the one of a wild species as H^ch^DR does. ABH^ch^R also showed a great capacity to regrow new shoots. All the florets were sterile.

#### 2.1.3. Amphiploid AABBH^ch^H^ch^RR

Hybrid H^ch^DR and the two ABH^ch^R hybrids were treated with colchicine to induce WGD and obtain the corresponding amphiploids. After multiple attempts with both hybrids, only the ABH^ch^R using tritordeum HT377 was duplicated. Seven duplicated ABH^ch^R seeds were obtained. The chromosome number of the obtained amphiploids AABBH^ch^H^ch^RR ranged from 49 to 54 chromosomes. None of the amphiploids had the complete set of 56 chromosomes. All the seeds germinated and became adult plants; however, out of the seven plants, only two of them produced progeny: one plant with 53 chromosomes produced 18 seeds, and another one with 51 chromosomes produced four seeds. We called these amphiploids AABBH^ch^H^ch^RR-1 and AABBH^ch^H^ch^RR-2, respectively. All the seeds appeared small and shriveled, and only nine seeds from AABBH^ch^H^ch^RR-1 and two seeds from AABBH^ch^H^ch^RR-2 germinated. For this study we analyzed three descendants of the AABBH^ch^H^ch^RR-1 amphiploid and two descendants of AABBH^ch^H^ch^RR-2.

Root tip metaphase spreads were analyzed by multicolour GISH (using the same genomic probe combination as with the ABH^ch^R hybrid) to establish the genome constitution of all the amphiploids obtained (Table 1). All individuals analyzed were aneuploids, with chromosome number ranging from 46 plus two telosomic chromosomes (46 + 2t) to 51 chromosomes (Figure 2c; Figure S1). The *H. chilense* genome was the most affected, with only seven chromosomes in some of the amphiploids (instead of the 14 chromosomes expected). All amphiploids showed at least one T1RS·1BL translocation coming from tritordeum HT377. There were also several telosomic chromosomes from durum wheat, rye, and *H. chilense*. Interestingly, AABBH^ch^H^ch^RR-2-2 possessed a centromeric translocation between rye and *H. chilense* (TH^ch^·R) (enlarged in Figure 2c); however, the rye chromosome arm involved in the translocation does not correspond to any of the chromosomes present in wild type rye. In rye, the nucleolar organizer region (NOR) is in the short arm of chromosome 1 (1RS) and it can sometimes be easily observed at metaphase I as a less stained band due to the more decondensed stage of this region (see the T1RS·1BL chromosome in Figure 2c). However, the rye chromosome translocated with the *H. chilense* one is a long rye arm with a very clear NOR closer to the centromere instead of the telomere. In wild type rye, no rye chromosome has this structure; therefore, this is a newly reorganized rye chromosome that originated in this material. A rye chromosome with the same reorganized long arm can also be observed in Figure 2c (enlarged). We could identify this reorganized chromosome because of the presence of the NOR in the non-canonical location, but this means that there are probably many more chromosome reorganizations between the chromosomes of each genome which we could not detect by GISH. Although we detected several structural rearrangements, none of the amphiploids presented any inter-genomic chromosome exchange, which suggests that recombination was still limited to homologous chromosomes. Having said that, all amphiploids had only undergone two rounds of meiosis after synthesis, so even if recombination between non-homologous chromosomes was possible, there were not many chances of recovering this recombination event in these five amphiploids. A higher number of plants or more generations would be necessary to confirm this observation.

The morphology of the partial amphiploids was similar to that of the ABH^ch^R hybrids but shorter. Each individual amphiploid was slightly different from each other due to the different chromosome composition, showing high diversity in height (Figure S2). For example, AABBH^ch^H^ch^RR-1-1 displayed a clearly stunted phenotype (Figure 3d) with compact spikes, while AABBH^ch^H^ch^RR-2-1 showed a much slimmer phenotype, both in vegetative growth as in spike development (Figure 3c). Unfortunately, all the florets were sterile. Aneuploids are frequently male sterile but still show some degree of female fertility; so with that in mind, we tried to pollinate the amphiploids with hexaploid wheat *T. aesticum* cv. Chinese Spring pollen in an attempt to recover some seeds. Unfortunately, no seed was ever set.

### 2.2. GISH Analysis of Meiotic Metaphase I Configuration in Hybrids and Amphiploids

#### 2.2.1. Meiotic Metaphase I Configuration in the Trigeneric Hybrid H^ch^DR

In diploid species, only homologous chromosomes recombine during meiosis to ensure accurate chromosome segregation. However, no homologous chromosomes are present in the haploid hybrid. Once we established that H^ch^DR was a euploid hybrid, we used GISH to study the meiotic behavior of the three genome’s chromosomes at metaphase I, using the same labelling conditions as we did for the somatic cells. To establish the level of recombination, we used chiasmata or crossover (CO) counting at meiotic metaphase I, which is the most common method of crossing over scoring. We were not able to demonstrate that all the chromosome associations observed were the result of a CO, i.e., chiasmatic, because this hybrid was sterile, and we could not recover the results of these recombination events in the next generation. For that reason, we decided to use the word “association” instead of CO number.

**Figure 4:**
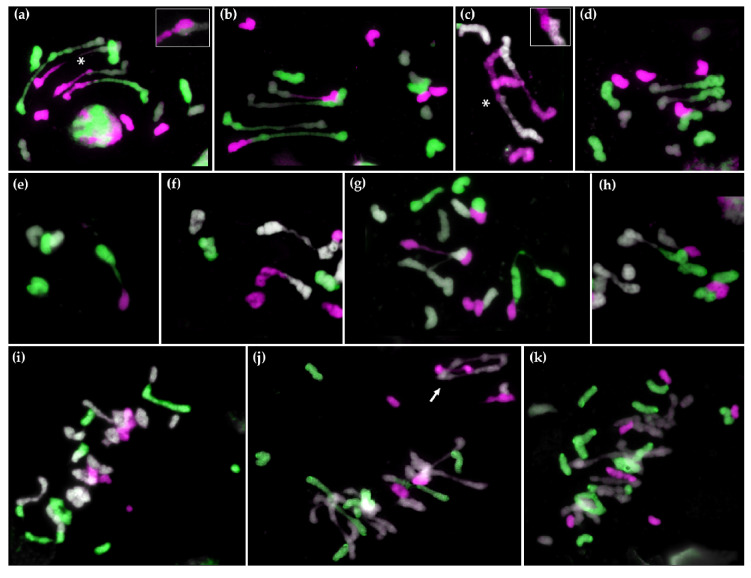
GISH of meiotic metaphase I configuration in the trigeneric hybrids H^ch^DR (**a**–**d**), ABH^ch^R (**e**–**h**), and aneuploid amphiploid AABBH^ch^H^ch^RR (**i**–**k**). *Hordeum chilense* is shown in magenta and rye in green. In H^ch^DR, *Aegilops tauschii* is shown in grey. In ABH^ch^R and AABBH^ch^H^ch^RR, durum wheat is shown in grey. In (**a**,**c**) a CO structure between *Hordeum* and *Aegilops* is indicated by an asterisk (*) and is enlarged. In (**j**) a wheat trivalent is indicated by an arrow.

A total of 134 meiotic metaphase I cells were analyzed using GISH (Table 2; Figure 4). Surprisingly, 75.37% of the cells (101 cells) showed chromosome associations. Most of these associations were rod bivalents between two chromosomes, but several ring bivalents and trivalents were also observed. One might expect that most of these associations were between chromosomes belonging to the same genome; however, that was not the case, with a higher number of associations observed between *Hordeum* and *Aegilops* (44.8%) followed by *Aegilops* and *Secale* associations (28.6%). We could even detect several trivalents where chromosomes from the three genera were involved. These results highlight the potential use of this material to promote recombination between genomes that would not normally recombine in a wild type situation. As already mentioned, we were not able to demonstrate that all the associations observed are quiasmatic because this hybrid is sterile; however, even if some of the more end-to-end associations could be non-chiasmatic, there are some very clear examples where the CO structure was clearly observed between two pairs of chromosome (Figure 4a,c), confirming recombination between the different genera. For that reason, we also included the number of associations where a clear CO was established in Table 2, which provides a very conservative estimation of the number of recombination events detected. As with the total number of associations, a higher number of COs was observed between *H. chilense* and *Ae. tauschii*.

#### 2.2.2. Meiotic Metaphase I Configuration in the Trigeneric Hybrid ABH^ch^R and its Corresponding Amphiploid AABBH^ch^H^ch^RR

A total of 147 meiotic metaphase I cells were analyzed, out of which 47 cells (40.8%) showed some chromosome association (Table 3). Although associations were observed between all the different genomes (Figure 4e–h), only rod bivalent structures were detected. The majority of the associations were between *Triticum* chromosomes (41%), followed by *Triticum* and *Hordeum* (20.5%), and *Triticum* and *Secale* (15.7%). Rod bivalents have one association per bivalent instead of the two associations per trivalent and ring bivalent structures, which emphasizes the lower number of associations observed in the ABH^ch^R hybrid compared with H^ch^DR. This could be explained by the presence of the *Ph1* locus on the 5B genome, which controls homologous recombination in wheat (see discussion). Moreover, not only were the number of associations lower, but also, contrary to the associations in H^ch^DR, the associations observed in the ABH^ch^R hybrid were extremely distal and thus, it is possible that they are non-chiasmatic. Only six of the 83 chromosome associations detected have a clear CO structure, indicating that they are chiasmatic. Unfortunately, since ABH^ch^R is also sterile, we cannot check the progeny to determine the output of the meiotic associations

Finally, we analyzed the meiotic behavior of the duplicated ABH^ch^R, the amphiploid AABBH^ch^H^ch^RR. As already mentioned, all the amphiploids obtained were aneuploid and had a different chromosome composition. For the meiotic studies we selected two of these amphiploids: AABBH^ch^H^ch^RR-1-1 (2n = 4× = 46 + 2t) and AABBH^ch^H^ch^RR-2-1 (2n = 48 + t). A total of 125 and 112 PMCs were analyzed from each genotype. Interestingly, despite being aneuploids and presenting multiple chromosome organizations, almost no chromosome association was observed between *Triticum*, *Hordeum,* and *Secale*. The presence of associations between the different genera was anecdotical. There were multiple rod and ring bivalents as expected in an amphiploid, but trivalents and quatrivalent were also present due to their aneuploidy (Figure 4j). No difference in terms of associations was observed between AABBH^ch^H^ch^RR-1-1 and AABBH^ch^H^ch^RR-2-1.

#### 2.2.3. Synaptonemal Complex Formation in H^ch^DR and ABH^ch^R Hybrids, and the Amphiploid ABBH^ch^H^ch^RR

During meiosis, homologous chromosomes gradually align along their length as a proteinaceous structure, and the synaptonemal complex (SC) forms between them. This process is known as “synapsis”, which will be completed at the pachytene stage of meiosis. Within this framework, homologs can recombine. After pachytene, the SC is degraded, while COs or chiasmata enable the homologs to remain associated at metaphase I and so segregate properly. SC formation is thus a prerequisite for CO formation and recombination in most species including wheat, rye, and barley. With this in mind, we decided to follow the process of synapsis in the two hybrids and amphiploids obtained in this work and check whether the lower number of associations observed in the ABH^ch^R hybrid was related to the process of synapsis. To follow the progression of synapsis we used antibodies against two of the main structural proteins that form the SC: ASY1 [24] and ZYP1 [25]. ASY1 is part of the lateral element of the SC, being loaded before synapsis; and in wheat, it will be observed in the chromosome regions that are not synapsed [26]. ZYP1 is part of the central region, which assembles between the lateral elements, and thus, it is present only in chromosome regions that are synapsed. In a diploid wild type situation, only homologous chromosomes are synapsed at pachytene; however, in polyploids such as wheat, SC formation can also occur between non-homologs under particular conditions such as the absence of homologs [26,27,28]. This is the situation of the haploid hybrids presented in this study; therefore, we expected to detect some level of synapsis in both H^ch^DR and ABH^ch^R hybrids.

**Figure 5:**
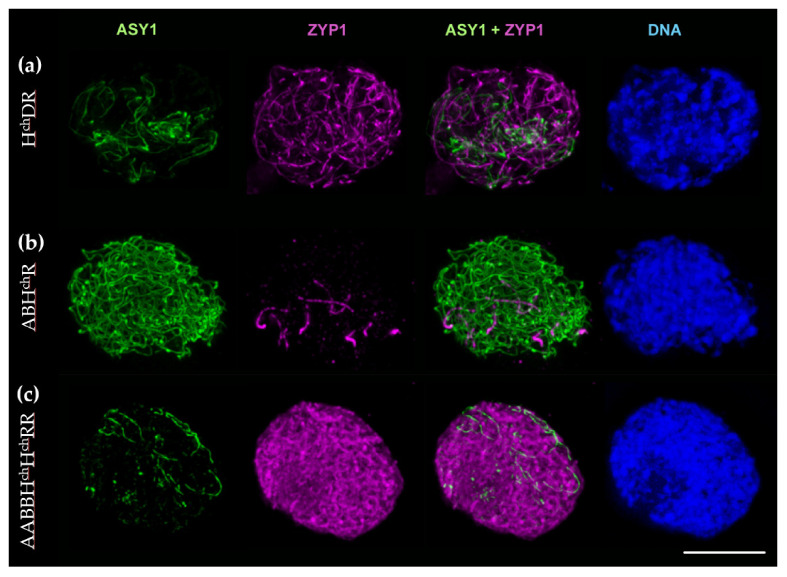
Immunolocalization of meiotic proteins ASY1 (green) and ZYP1 (magenta) in meiocytes from the trigeneric hybrids H^ch^DR (**a**) and ABH^ch^R (**b**), and the aneuploid amphiploid AABBH^ch^H^ch^RR (**c**). DAPI staining in blue. Scale bar, 10 μm.

We have shown that the H^ch^DR hybrid presented a high level of chromosome associations and CO formation in this work, and as expected, a high level of synapsis was detected in this hybrid (Figure 5a). There was a different level of synapsis amongst meiocytes, but overall, the level of synapsed regions (labelled by ZYP1 in magenta) was greater than the non-synapsed regions (labelled by ASY1 in green). This indicates that non-homologous chromosomes are able to synapse to a great extent. The situation of the ABH^ch^R hybrid was rather different. As mentioned before, we also observed chromosome associations between the different genomes in this hybrid; however, most associations were extremely distal, and we could not be sure that they would lead to any recombination event. The results of the immunolabelling support this suspicion, since almost no synapsis was detected in these hybrids (Figure 5b). Finally, we followed the process of synapsis in the aneuploid amphiploids AABBH^ch^H^ch^RR. Since almost the complete set of homologs was present in these amphiploids, most of the chromosomes were synapsed. However, synapsis was never complete and, although minimal, some ASY1 labelling was always observed in all meiocytes (Figure 5c) as a consequence of the aneuploid nature of these hybrids.

## 3. Discussion

Distant hybridization, the process of making crosses between different species or genera, is one the methods that has been successfully exploited in the *Triticeae* tribe to enrich the genetic gene pool of cultivated crops (and particularly wheat) by exploiting the genetic diversity present in wild relatives. However, the transfer of alien chromatin is not straightforward due to the presence of several reproductive barriers that hinder the genetic transfer among them. These include hybridization incompatibilities due to sterility, and more importantly, suppressed recombination between the crop and the wild relative. The more evolutionary distant the species to be crossed, the more chances there are to show hybridization incompatibilities and a lack of recombination. In the case of polyploid wheat, the lack of meiotic recombination between wheat and related species will not only depend on syntheny, but also on a system that appeared during wheat polyploidization to stabilize the wheat genome during meiosis and that has been mainly attributed to the *Ph1* (*Pairing homoeologous 1*) locus [29,30]. This locus located on chromosome 5B ensures that only identical chromosomes (homologs) crossover and recombine during meiosis, and none of the very similar chromosomes (homeologs) present in wheat. This locus will thus prevent crossover and recombination between wheat and related species. Recently, this phenotype effect on recombination has been attributed to the meiotic gene *ZIP4* (*TaZIP4-B2*) [28,31,32], although the exact mechanism of how it works remains a mystery.

In this work, we used distant hybridization to obtain two hybrids, each one formed by species from three different genera: *Hordeum-Aegilops-Secale* (H^ch^DR) and *Triticum-Hordeum-Secale* (ABH^ch^R), and a trigeneric amphiploid (AABBH^ch^H^ch^RR). Both hybrids were sterile, which was expected due to the evolutionary distance between these three genera, and in the case of H^ch^DR, further expected due to the trihaploid nature of the hybrid.

### 3.1. CO Formation Can Take Place between Ae. tauschii, Barley and Rye in the H^ch^DR hybrid

This was not the first time that an H^ch^DR hybrid was obtained. In 1992, Cabrera and Martín [33] reported the production of this hybrid, with the only difference that the *H. chilense* accession used in their work was the accession H1, while in the present work, we used the accession H7. They did not observe any meiotic chromosome association, reporting no CO formation in this hybrid, and they could not produce the corresponding amphiploid either. Three genetically different ecotypes have been described among *H. chilense* species [34], with H1 belonging to ecotype I and H7 to ecotype II. Interestingly, the cytoplasm of *H. chilense* accession H1 produced wheat male sterility, while the one of H7 produced perfectly fertile lines [35,36]. Thus, we decided to repeat this hybrid but using the accession H7 of *H. chilense*. Surprisingly, we discovered plenty of associations and CO formation in this new hybrid. Most of the associations were between chromosomes from different genera instead of between chromosomes from the same genome. This is probably due to the cereal synteny [37], which suggests that these associations were between homologous chromosomes. Chiasma or CO counting at meiotic metaphase I is the most common and straight forward method of scoring crossing-over events, particularly in organisms in which genetic analysis is difficult or impossible to perform, as is the case of our hybrids. However, it has also been reported that very distal end-to-end chromosome associations might not be chiasmatic and are a remnant of prophase pairing [38]. It is for that reason that in this work, we decided to be cautious and use the more general word “association” instead of CO number. However, some of the associations showed such a clear CO structure that we felt confident to also provide a number of COs in the results section. This number would provide a minimal number of recombination events, though most probably, the quantity is considerably higher. Most associations and CO formation occur between *H. chilense*-*Ae. tauschii* followed by *S. cereale*-*Ae. tauschii*, which suggest homology between these *Triticeae* species. As already mentioned, synaptonemal complex (SC) formation is a prerequisite for CO formation and recombination in wheat, rye, and barley. Thus, we used immunolabelling to follow the process of synapsis in the H^ch^DR hybrid. In agreement with the level of recombination detected, a high level of synapsis was observed in this hybrid.

Previous studies have shown chromosome association between *H. chilense*-*Ae. tauschii* [21,39] and to a very low extent, between *H. chilense* and *S. cereale* chromosomes [40]. Unluckily, none of these authors mention in their work the specific accession of *H. chilense* and/or *Ae. tauschii* and *S. cereale* used, so we can only speculate that this could be a reason for the contradictory results reported. Some of these accessions might possess some *locus* or more probable specific alleles that promote or suppress recombination between non-homologous chromosomes. Rye in particular is known to have a polygenic system that controls the pairing between homoeologous chromosomes of wheat [41,42]. There are also many different factors that have been reported to affect CO formation in several species such as growing temperature, photoperiod conditions or nutritional availability amongst others (reviewed in [32,43]). This could provide an explanation for the differences observed among studies, while highlighting the importance of considering these factors when performing distant hybridization.

### 3.2. Most Associations between Wheat, Rye, and Barley in the ABH^ch^R Hybrid Are Probably Achiasmatic

Several intergeneric hybrids between species of the genus *Hordeum* with species of the genus *Triticum* and *Secale* have been produced in the past [42,44,45]. In all these cases, triticale (the hybrid between wheat and rye) was used to obtain the trigeneric hybrid. When meiotic analysis was performed in these studies, the number of associations observed between the different genomes was insignificant (with a maximum of 0.15 associations per cell), and the corresponding amphiploid was never obtained. We attempted to produce this trigeneric hybrid using a different approach. Many haploid hybrids have been obtained between *Hordeum* and *Triticum* (reviewed in [15]), but the first *Hordeum-Triticum* amphiploid produced (tritordeum), and the only one that managed to turn into a crop, was the one using *H. chilense* as the mother species [5]. It is for that reason that we have used the wild barley *H. chilense* and tritordeum (instead of triticale) in this new approach. As hoped, we found a higher number of chromosome associations than those found in preceding reports. The absence of GISH in the former *Hordeum-Triticum* hybrids studies prevented the identification of the genomes involved in the associations. Here, we identified a mean of 0.56 associations per cell, from which almost half (0.23 associations) were between *Triticum* chromosomes, followed by *Triticum-Hordeum* (0.12) and *Triticum-Secale* (0.09). We can presume that the high number of associations between *Triticum* was the result of associations between homeologs of the A and B genomes, but further analysis would be needed to confirm this assumption. However, there was a great difference between the type of associations observed in this hybrid and the ones observed in the H^ch^DR hybrid. Most of the associations in ABH^ch^R were extremely distal, reminding us of those described previously as non-chiasmatic [38] and being a remnant of meiotic prophase I. As with the H^ch^DR hybrid, we counted those associations showing a clear CO structure, resulting in only six COs in 147 cells (0.04 COs per cell). This result, although disappointing, was not a surprise. As already mentioned, the *Ph1* locus on chromosome 5B would prevent recombination between wheat homeologous chromosomes and between wheat and related species. We were hoping that non-wheat chromosomes were still allowed to recombine; however, the results obtained here suggest that *Ph1* could also prevent recombination between the *Hordeum* and *Secale* chromosomes present within the same nucleus. However, the answer might not be so simple. As we did with H^ch^DR, we followed the process of synapsis in the ABH^ch^R hybrid. Surprisingly, almost no synapsis was detected in this hybrid. This result was shocking because in hexaploid wheat-rye hybrids (ABR), the level of synapsis at pachytene was reported as 26–27% whether *Ph1* was present or absent [26]. Thus, we do not think that this lack of synapsis is related to the presence of *Ph1*. Synapsis is a prerequisite for CO formation in cereals; however, it is common to observe synapsis even when no CO formation can take place [26,27,46]. All studies mentioned were performed in hexaploid wheat, and therefore, in the presence of the D genome. Thus, it is possible that the D genome chromosomes are more involved in homeologous synapsis. On the other hand, different levels of synapsis have been reported in haploid hexaploid wheat depending on the variety studied, with 40% reported in haploid Chinese Spring vs. 90% in haploid Kedong [27]. So as with CO formation, there seems to be a great deal of allelic variation among different varieties or accessions in terms of homeologous synapsis. Although there are many meiotic studies reporting the number of homeologous COs, not many studies have tackled the study of the synaptonemal complex apart from those already mentioned in this work. Further studies in wheat and other *Triticeae* species will be required to clarify these results. In any case, the fact that there is almost no synapsis corroborates the assumption of the non-chiasmatic character of the associations observed in this hybrid.

### 3.3. The Amphiploid AABBH^ch^H^ch^RR Is Highly Unstable and Mostly Sterile

Multiple synthetic trigeneric hybrids combining species from the *Triticeae* tribe have been obtained; however, the development of the corresponding synthetic amphiploid is rarely reported. Durum wheat-*Thinopyrum-Lophopyrum* hybrids did result in amphiploids [47,48], but to our knowledge, no other trigeneric amphiploid have been described in the literature. During this study, we repeatedly used colchicine treatment to duplicate the genome of the H^ch^DR and the ABH^ch^R hybrids. We finally succeeded in the duplication of the ABH^ch^R hybrid obtained using tritordeum line HT377 (with the translocation T1RS·1BL). We cannot provide any explanation for the success of this specific line. The durum wheat-*Thinopyrum-Lophopyrum* hybrid and ABH^ch^R both share the durum wheat genome and durum wheat cytoplasm, so one might think that there could be something in the already polyploid durum wheat that facilitates WGD. However, this would not explain why only the ABH^ch^R hybrid using HT377 was duplicated. These two tritordeums are advanced lines, and as such, both have wheat cytoplasm. HT377 has a T1RS·1BL translocation that is not present in HT474; but this is only one of the multiple differences between these two lines. Both HT377 and HT474 are advanced breeding lines with a complex pedigree behind them, and any of the multiple lines and varieties used in their production could be related to the duplication success. Hybridization and polyploidization are complex processes that trigger a series of genetic and genomic mechanisms including the genesis of novel structural variations such as translocations and inversions, homeologous exchanges, transposable elements mobilization, and novel insertional effects amongst others [49]. This stress exerted on the new polyploid, whose genomes are placed together within a single nucleus, was referred to by Barbara McClintock as genomic shock [50]; however, although widely accepted, it is a phenomenon far from being understood.

Feldman and Levi [2] propose that allopolyploidization accelerates genome evolution in two ways: through “revolutionary” changes that arise shortly after genomes are merged, versus “evolutionary” changes that occur more gradually over time. We could detect some of these revolutionary changes in our newly synthesized amphiploids. None of the seven duplicated seeds obtained had the complete set of 56 chromosomes; they were all aneuploid AABBH^ch^H^ch^RR with a chromosome number between 49 and 54. All seven seeds became adult plants but only two of them were fertile and produced progeny: one plant with 53 chromosomes (AABBH^ch^H^ch^RR-1) and another one with 51 chromosomes (AABBH^ch^H^ch^RR-2). For this study, we examined the progeny of these two aneuploid amphiploids. Remarkably, all the amphiploids analyzed showed multiple structural variations, and in all cases but one, the chromosome number was lower than in the parental line. This chromosome elimination was not random, *H. chilense* being the genome more clearly affected. Interestingly, primary octoploid triticale (the synthetic amphiploid between hexaploid wheat and rye, AABBDDRR) is meiotically unstable with variable frequency of univalents in metaphase I and reduced fertility [51,52,53,54,55]. This meiotic instability leads to the production of aneuploid progeny with rye chromosomes mostly being the cause of aneuploidy [55,56,57,58] and in some extreme cases, being able to eliminate all rye chromosome and revert to hexaploid wheat [59]. It has been speculated that late DNA replication of rye heterochromatin interferes with chromosome synapsis when rye chromosomes are placed in a wheat genetic background [60,61,62]; however, in the case of the AABBH^ch^H^ch^RR presented here, *H. chilense* was the genome more affected, but also the smaller genome and the one with lower level of heterochromatin. Thus, we need to find further explanations for its preferential elimination. Apart from aneuploidy, other structural rearrangements were detected in these amphiploids. A centromeric translocation between rye and *H. chilense* (TH^ch^·R) was identified, where the rye chromosome arm involved in the translocation does not correspond to any of the chromosomes present in wild type rye. We could identify this reorganized chromosome because of the presence of the NOR in the non-canonical location, but this means that there are probably many more chromosome reorganizations within the chromosomes of the same genome that we cannot detect by GISH. We frequently focused on meiotic recombination, but distant hybridization is also known to trigger reorganizations of the different genomes [63,64,65], which can be extremely useful in breeding and have often been used to incorporate desirable traits from relatives into wheat [66,67]. None of the new amphiploids obtained presented any inter-genomic chromosome exchange, which suggests that the presence of *Ph1* restricted recombination to homologous chromosomes. Nevertheless, this was the first generation of the amphiploids after synthesis, and thus, they had only undergone two rounds of meiosis. A higher number of plants or more generations would be necessary to confirm this observation.

## 4. Materials and Methods

### 4.1. Plant Material

The plant material used in this study and its production is described in Figure 1. It includes the wild barley *Hordeum chilense* accession H7 (2n = 2× = 14; genome H^ch^H^ch^); *Aegilops tauschii* (2n = 2× = 14; genome DD); *Secale cereale* (accession BGE003054 from the Centro de Recursos Fitogenéticos INIA, Alcalá de Henares, Spain) (2n = 2× = 14; genome RR); and *×Tritordeum martinii* lines HT474 (2n = 6× = 42; genome AABBH^ch^H^ch^) and HT377 (with the translocation T1RS·1BL). All plant material was provided by Prof. Antonio Martín from the collection maintained at the Institute for Sustainable Agriculture in Córdoba (Spain).

The production of the trigeneric hybrid between [*Ae. tauschii × H. chilense*] × *S. cereale* was performed as described in [33,68]. Briefly, the chromosome number of *H. chilense* and *Ae. tauschii* was doubled using colchicine according to the capping technique [57,69]. The generated tetraploid *H. chilense* (2n = 4× = 28, H^ch^H^ch^H^ch^H^ch^) was pollinated with the tetraploid *Ae. tauschii* (2n = 4× = 28, DDDD), and giberellic acid at a concentration of 75 ppm was applied to pollinated florets 24 h after pollination. Three weeks later, embryos were rescued and cultured in half-strength (MS/2) Murashige and Skoog medium [70] supplemented with 30 g/L of sucrose and 10 g/L of agar. When plants reached the three-leaf stage, they were transplanted into pots. Finally, the *H. chilense* × *Ae. tauschii* amphiploid was pollinated with *S. cereale*, and the embryo rescued, as mentioned before, to produce the trigeneric hybrid H^ch^DR (n = 3× = 21).

To produce the trigeneric hybrid [*T. turgidum* × *H. chilense*] × *S. cereale*, we used hexaploid *×Tritordeum martinii* (lines HT377 and HT474) to speed up the breeding process. Hexaploid tritordeum is the amphiploid between *T. durum* (2n = 4× = 28; AABB) and *H. chilense;* thus, by crossing it as a female parent with *S. cereale,* we could immediately obtain the trigeneric hybrid ABH^ch^R (n = 4× = 28). We obtained a hybrid with each of the tritordeum lines. In this case, embryo rescue was not necessary to obtain the hybrids.

We used colchicine in an attempt to double the genome of the H^ch^DR and the two ABH^ch^R hybrids to obtain the corresponding amphiploids. Only the ABH^ch^R hybrids using tritordeum HT377 could be duplicated and the corresponding amphiploid obtained (2n = 8× = 49–54, AABBH^ch^H^ch^RR).

Somatic chromosome number was counted for every new genomic combination obtained (as described in the next subsection) to confirm the genome constitution.

### 4.2. Genomic In Situ Hybridization (GISH) of Mitotic and Meiotic Cells

All plants were grown in a controlled environment with a 16 h light/8 h night photoperiod at 20 °C day and 15 °C night, with 70% humidity. For meiotic studies, tillers were harvested at early booting stage when the flag leaf ligule was just visible. For each dissected floret, one of the three synchronized anthers was squashed in 45% acetic acid in water to identify the meiotic stage. If they were at metaphase I, the two remaining anthers were fixed in 100% ethanol/acetic acid 3:1 (*v*/*v*) and kept at 4 °C until needed. For preparation of meiotic metaphase I chromosome spreads, each fixed anther was placed on a glass slide with a few drops of 45% acetic acid and squashed under a cover slip. The cover slip was removed by immersing the slide in liquid nitrogen for a few seconds and removing it with a quick movement. Slides were air-dried and kept at 4 °C until GISH. The preparation of mitotic metaphase spreads was carried out as described previously [71,72]. Briefly, excised root tips were treated with nitrous oxide gas to accumulate metaphase I cells and later digested in 1% pectolyase Y23 and 4% cellulose Onozuka R-10 (Yakult Pharmaceutical, Tokyo, Japan) solution in 1× citrate buffer.

Genomic *in situ* hybridization (GISH) was performed as previously described [57]. Briefly, genomic DNA from *H. chilense* (H^ch^ genome) and *S. cereale* (R genome) was used as probes. *T. turgidum spp. durum* (AB genome) genomic DNA was used as a competitor in the hybridization mix. *H. chilense* and *S. cereale* genomes were labeled with biotin-16-dUTP and digoxigenin-11-dUTP using the Biotin- or the DIG-nick translation mix, respectively, according to the manufacturer’s instructions (Sigma, St. Louis, MO, USA). Biotin-labeled probes were detected with Streptavidin-Cy3 or Streptavidin-Cy5; digoxigenin-labeled probes were detected with anti-digoxigenin-FITC (Sigma, St. Louis, MO, USA).

### 4.3. Immunolocalisation of Meiotic Proteins

Identification of the desired meiotic stages was performed as described above for the meiotic metaphase I, only that for immunolocalization studies, anthers were fixed in paraformaldehyde 4% for 15 min and processed immediately. Meiocytes of the trigeneric hybrids H^ch^DR and ABH^ch^R, and of the amphiploid AABBH^ch^H^ch^RR were embedded in acrylamide pads to preserve their 3D structure as described previously [26]. Immunocalisation of meiotic proteins ASY and ZYP1 was performed as described previously [28] using Anti-TaASY1 [73] raised in rabbit and used at a dilution of 1:250 and anti-HvZYP1 [74] raised in rat and used at a dilution of 1:200. Anti-rabbit Alexa Fluor^®^ 488 and anti-rat Alexa Fluor^®^ 568 (invitrogen) were used as secondary antibodies. Pads were counterstained with DAPI (1 μg/mL).

### 4.4. Image Acquisition and Analysis

Hybridization signals in mitotic and meiotic metaphase I spreads were examined using a Leica DM5500B microscope equipped with a Hamamatsu ORCA-FLASH4.0 camera and controlled by Leica LAS X software v2.0. Digital images were processed using Adobe Photoshop CS5 (Adobe Systems Inc., San Jose, CA, USA) extended version 12.0 × 64. Polyacrylamide-embedded meiocytes were optically sectioned using the same Leica DM5500B microscope. Z-stacks were processed using the deconvolution module of the Leica LAS X Software package. Images were processed using Fiji (an implementation of ImageJ, a public domain program by W. Rasband available from http://rsb.info.nih.gov/ij/).

## 5. Conclusions

The study of hybridization and polyploidization in *Triticeae* is an excellent example of the integration of more basic research studies with their application in plant breeding. The results presented in this work demonstrate that recombination between the genera of three economically important crops is possible. However, this study also highlights the complexity and the multiple factors involved in the success of a particular distant hybridization event in the transfer/exchange of genetic variability among species. The production of haploid hybrids, the subsequent WGD, the stability and fertility of the obtained amphiploid, and of course, inter-genome recombination, are all highly affected by abiotic factors, but maybe even more importantly, they seem to depend on a substantial amount of allelic variation among accessions and varieties within a species. Much more work is still needed to understand how all of these factors exert their effect and how they interact between each other. A better understanding of these interactions would definitively translate into improved manipulation methods for crop breeding.

## Figures and Tables

**Table 1:** Chromosome configuration of the five aneuploid amphiploids AABBH^ch^H^ch^RR obtained in this work. Telosomic chromosomes are abbreviated as “t”.

Line	Chromosome Number	Chromosome Configuration			Centromeric Translocation
		AB Genome	H^ch^ Genome	R Genome	
AABBH^ch^H^ch^RR-1-1	46 + 2 t	27 + 2 arms	8 + 1 t	9 + 2 arms + 1 t	2 TR·AB
AABBH^ch^H^ch^RR-1-2	51	28 + 2 arms	10	11 + 2 arms	2 TR·AB
AABBH^ch^H^ch^RR-1-3	49	28 + 1 arm	9	11 + 1 arm	1 TR·AB
AABBH^ch^H^ch^RR-2-1	47 + 1 t	25 + 2 arms + 1 t	7	13 + 2 arms	2 TR·AB
AABBH^ch^H^ch^RR-2-2	51	27 + 2 arms	7 + 1 arm	14 + 3 arms	2 TR·AB + 1 TH^ch^·R

**Table 2:** Number of chromosome associations observed in 134 cells of the trigeneric hybrid H^ch^DR. In the right column, the number of associations with a clear crossover structure is also shown.

	No. and Type of Chromosome Associations			Total % of Associations	Crossover	
	Rod Bivalent	Ring Bivalent	Trivalent		No.	%
*Hordeum-Hordeum*	13	-	-	6.2	2	2.8
*Aegilops-Aegilops*	4	-	-	1.9	-	-
*Secale-Secale*	12	-	-	5.7	3	4.2
*Hordeum-Aegilops*	84	5	-	44.8	33	46.5
*Hordeum-Secale*	15	-	-	7.1	7	9.9
*Aegilops-Secale*	60	-	-	28.6	23	32.4
*Hordeum-Aegilops-Secale*	-	-	3	2.9	3	4.2
*Hordeum-Aegilops-Aegilops*	-	-	1	1.0	-	-
*Hordeum-Secale-Secale*	-	-	1	1.0	-	-
*Hordeum-Hordeum-Aegilops*	-	-	1	1.0	-	-
	188	10	12		71	

**Table 3:** Number of chromosome associations observed in 147 cells of the trigeneric hybrid ABH^ch^R.

	Rod Bivalent	Total % of Associations	No. COs
*Triticum-Triticum*	34	41.0	1
*Hordeum-Hordeum*	6	7.2	0
*Secale-Secale*	9	10.8	2
*Triticum-Hordeum*	17	20.5	0
*Triticum-Secale*	13	15.7	2
*Hordeum-Secale*	4	4.8	1
Total No. Associations	83		6

## Data Availability

No new data were created or analyzed in this study. Data sharing is not applicable to this article.

## Supplementary Materials

The following are available online at https://www.mdpi.com/2223-7747/10/1/113/s1, Figure S1: Root-tip metaphases of the aneuploid amphiploids (a) AABBHchHchRR-1-1, (b) AABBHchHchRR-1-2, (c) AABBHchHchRR-1-3, and (d) AABBHchHchRR-2-2. H. chilense (barley) is shown in magenta, S. cereale (rye) in green and T. turgidum spp. durum (durum wheat) in grey. Figure S2: Adult plant and spike from the aneuploid amphiploids (a) AABBHchHchRR-1-2, (b) AABBHchHchRR-1-3, and (c) AABBHchHchRR-2-2.
