# Supplementary figures and images for: Wheat, Rye, and Barley Genomes Can Associate during Meiosis in Newly Synthesized Trigeneric Hybrids
Source: Plants (Basel). 2021 Jan 7;10(1):113. doi: 10.3390/plants10010113 (PMC7826760; doi:10.3390/plants10010113)

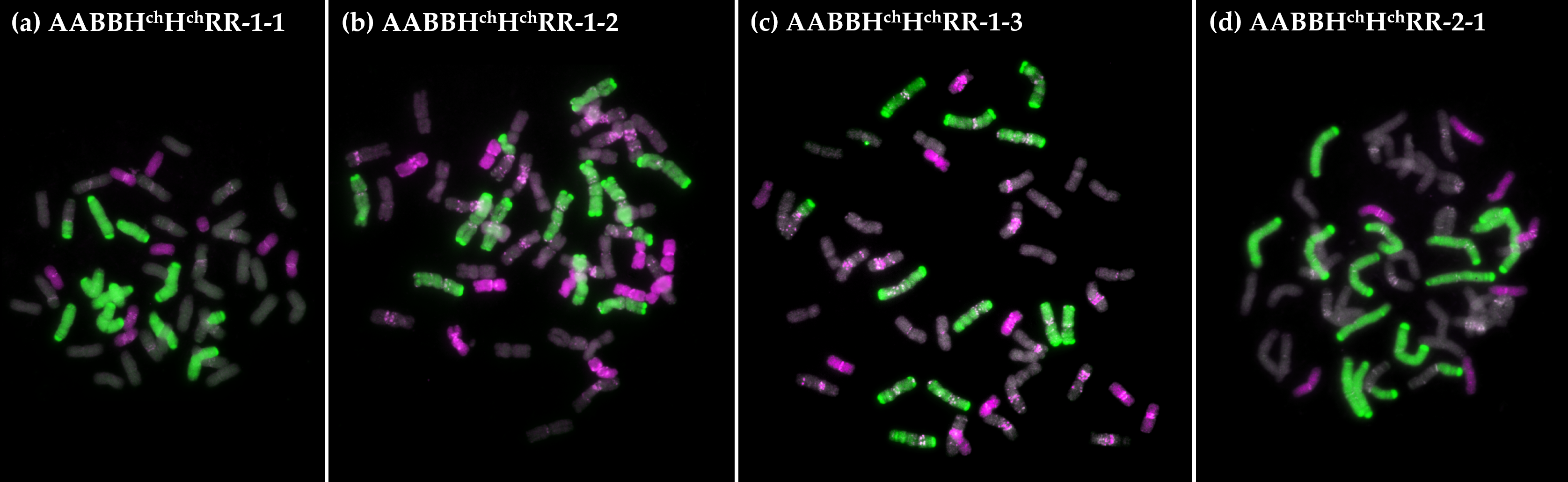

Supplement: Supplementary file 1 [file plants-10-00113-s001.zip › Figure S1.tif]

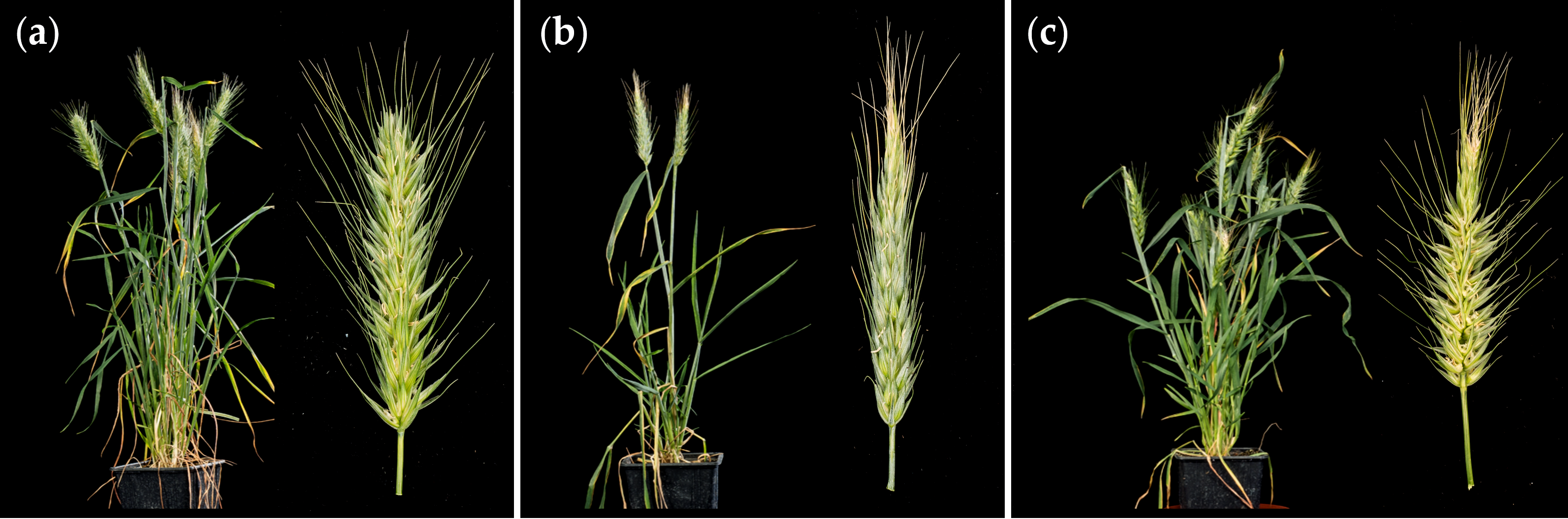

Supplement: Supplementary file 1 [file plants-10-00113-s001.zip › Figure S2.tif]
